# *Drosophila* immune priming against *Pseudomonas aeruginosa* is short-lasting and depends on cellular and humoral immunity

**DOI:** 10.12688/f1000research.2-76.v1

**Published:** 2013-03-05

**Authors:** Theodoulakis Christofi, Yiorgos Apidianakis

**Affiliations:** 1 Department of Biological Sciences, University of Cyprus, Nicosia, 1678, Cyprus

## Abstract

Immune responses are traditionally divided into the innate and the adaptive arm, both of which are present in vertebrates, while only the innate arm is found in invertebrates. Immune priming experiments in *Drosophila melanogaster* and other invertebrates during the last decade have challenged this dogma, questioning the boundaries between innate and adaptive immunity. Studies on repeated inoculation of *Drosophila* with microbes reveal a long-lasting cellular immunity adaptation against particular microorganisms. Here we study the lasting effect of immune priming against infection with *Pseudomonas aeruginosa*, an opportunistic human pathogen that is lethal to the common fruit fly. *Drosophila* priming with heat-killed or low in virulence *P. aeruginosa* extends fly survival during a secondary lethal infection with a virulent strain of the same species. The protective immune response can last for more than 10 days after exposure to a persistent low-in-virulence live infection, but it is eliminated 7 days after the host is primed with heat-killed bacteria. Moreover, not only the cellular, but also the systemic NF-κB-mediated immune responses contribute to immune priming. Thus each microbe might elicit different mechanisms of immune priming that may or may not last for long.

## Introduction

Organisms are targets of various infectious microbes that attack a host by penetrating its body in order to feed and reproduce. To cope with infection, each host has developed physical barriers that inhibit microbial entry and tissue homeostasis factors and immune responses that may increase tolerance or resistance to infection. Immune responses can directly target microbes and are observed in most species, from bacteria to mammals through a variety of versatile mechanisms that may be of a broad or of a very microbe-specific nature. In terms of immediacy and specificity, the immune responses have been traditionally divided into innate and adaptive ^1^.

### "Traditional" vertebrate innate and adaptive immunity

Defence barriers, such as the physical barrier of the skin or insect cuticle, intestinal mucus or insect peritrophic membrane and the low or high acidity in the gastrointestinal tract are the first lines of defence against invading microorganisms ^1^. In addition, innate immunity can be elicited as a fast and broad response against pathogens. Specialised immune cells such as macrophages and neutrophils can internalise and digest microbes initiating an inflammatory response at the site of infection or systemically to produce a hostile environment for the intruder. The complement group of proteins can also be activated to fight invading microorganisms ^2– 5^.

Components of the immune system can exhibit further specificity and acquire memory of past infections. This evolutionary step, termed ‘adaptive immunity’, can only be seen in vertebrates and displays antigenic specificity, diversity, immunologic memory and self/non-self recognition. Adaptive immunity depends on innate immune responses such as phagocytosis and inflammation that trigger the utilisation of specific immune response on the invader ^6^. Adaptive immunity can produce a variety of immune responses specific to antigenic challenges through a variety of effectors. Cooperation between lymphocytes and antigen presenting cells is the main mechanism of action, according to which naive B lymphocytes expressing a membrane-bound antibody molecule are activated when they bind to their specific antigen and divide quickly into memory B cells and effector B cells that induce humoral immunity ^7^. T lymphocytes recognise cell antigens only from major histocompatibility complex molecules and proliferate into memory and effector T cells. T lymphocytes can be subdivided into T helper (TH) and T cytotoxic (TC) cells that are responsible for the tight regulation of the immune response and cytotoxic T lymphocyte activity (CTL) ^8^. During a primary immune response, naive T and B lymphocytes become antigenically committed and expand rapidly in a process called clonal selection ^9^. Immunologic memory can be attributed to these memory cells, which have long life spans and exhibit a heightened response during secondary exposure.

### The immune response of *Drosophila melanogaster*

*Drosophila* is the main model organism for studying innate immunity among invertebrate species. *Drosophila* immune defences include physical barriers ^10^, homeostatic factors ^11^ and local and systemic immune responses. Three systemic responses have been described in the fly: the humoral response, melanization and the cellular response ^12^. Similarly to other arthropods, *Drosophila* contains a circulating hemolymph with blood cells called hemocytes. These can be sub-divided into three cell types with different functions: plasmatocytes, lamellocytes and crystal cells ^12^. Plasmatocytes, which comprise the majority of mature hemocytes, can clear unwanted cells and pathogens through phagocytosis ^12^. Lamellocytes can only be observed in larvae where they encapsulate and neutralise larger objects, and crystal cells are involved in the melanization process ^12^. The synthesis and deposition of melanin in the affected area is thought to play an important role in wound healing, captivation and encapsulation of invading microbes and production of toxic substances for subsequent microbial destruction ^12^. Coagulation occurs to prevent hemolymph loss but can also trap microorganisms and facilitate their destruction ^13^.

The *Drosophila* fat body is analogous to the mammalian liver where humoral response molecules are produced ^14^. Bacteria and fungi activate the Toll pathway indirectly via production of a “danger” signal ^15^. In addition, bacteria and fungi induce the Toll and Imd pathways directly by the recognition of bacterial peptidoglycan and fungal beta-glucan via peptidoglycan recognition proteins and Gram-negative binding protein 3 respectively ^16^. Upon systemic immune response Toll and Imd pathways induce the NF-κB factors Dif and Rel respectively, which in turn induce the expression of several antimicrobial peptides (AMP) ^17^. Besides AMPs, plasmatocytes locate and phagocytose bacteria through the help of scavenger receptors Eater and Dscam ^18, 19^. The epithelial barrier also exhibits local immunity where production of reactive oxygen species (ROS) and AMPs provides a defence mechanism in the gut ^20^. In addition to the plasmatocyte-expressed cytokine unpaired 3 (Upd3) induces the JAK/STAT pathway mediating robust responses to bacterial and fungal infection ^12^; while the same pathway can be induced upon tissue damage or viral infection ^21, 22^.

### Evidence of pathogen specific immunological memory

The aforementioned innate immune responses have not been proven to exhibit adaptive properties such as memory or specificity. However, the classic division between innate and adaptive immunity has recently been brought into question by a number of studies in invertebrate organisms, which challenge the currently defined boundaries of immunological memory ^23^.

Recent evidence suggests that arthropods can display selected 'specificity' towards particular microorganisms. Pham and colleagues demonstrated that the fruit fly exhibits a specific primed immune response dependent on plasmatocytes ^24^. They tested various pathogens including bacteria and fungi and found that flies mount a prolonged protective response against *Streptococcus pneumoniae* after being primed with a sub-lethal or heat-killed dose of the bacterium. *S. pneumoniae* bacteria are killed by the host within 1 day of infection only in primed flies whereas unprimed flies still contained bacteria indicating that survival depends on the elimination rate of *S. pneumoniae* ^24^. They also found a similar adaptation with the natural fungal pathogen *Beauveria bassiana*.

Protection against other bacteria was not observed by priming with *S. pneumonia*. Conversely, other heat-killed bacteria – known to be strong immune activators - did not exert a protective response against *S. pneumoniae*. Immune pathway mutants demonstrated that immune priming is due to the activation of the Toll pathway but not due to the expression of AMPs. These findings illustrate the selective adaptability of the immune system through the activation of Toll pathway and plasmatocytes. However it is important to note that not all pathogens respond in the same way. In this report, we use the example of *Pseudomonas aeruginosa*, a gram-negative bacterium that induces the Imd and the Toll pathways, as well as the cellular immune response.

### Immune priming with *P. aeruginosa*

Previous studies show that live *P. aeruginosa* infection with the low-in-virulence CF5 strain primes the immune system and helps to protect *Drosophila* from subsequent lethal infection with the virulent PA14 strain (UCBPP-PA14) ^25^. This protection is evident 6, 12 and 24 hours post immune priming and involves the activation of both the Imd and the Toll pathway ^25^. Here we assess the duration of this protective response and the involvement of humoral and cellular immune responses. We find that immune priming with heat-killed *P. aeruginosa* CF5 confers protection for less than 7 days and that the Imd and the Toll pathways, as well as phagocytosis, contribute to host protection at 2 and 5 days respectively post-immune priming.

## Methods

### Fly strains

Wild type Oregon R and Eater mutant flies were a gift from Christine Kocks ^18^. Canton S (CS) was obtained from Bloomington stock Center. Imd ^1^, Rel ^E20^ and Dif ^1^ mutant flies were a gift from Bruno Lemaitre.

### Bacterial strains and infection assays

*P. aeruginosa* strains PA14 and CF5 are previously described human isolates ^25^. For inoculation with live CF5 cells flies were pricked with a needle previously dipped into a solution of 3 x 10 ^8^ CF5 cells/ml or in PBS as a control as previously described ^25, 26^. For CF5 colony forming units (CFUs) enumeration, 3 flies per time point, in triplicates, were ground and plated every two days. Using the injection method 9.2 nl of a bacterial solution was introduced into the fly thorax to prime or infect the flies and host survival was measured every hour as previously described ^25, 26^. 9.2 nl of a solution containing 3 x 10 ^8^ heat-killed CF5 cells/ml or the equivalent volume of PBS was injected to prime flies with heat-killed *P. aeruginosa*. Primed flies were subsequently injected with 9.2 nl of a live bacteria solution containing 3 x 10 ^7^ PA14 cells/ml.

### Statistical analysis

Fly survival kinetics were analyzed using the MedCalc software ( www.medcalc.org/). Survival curve analyses were performed using the Logrank test of the Kaplan-Meier survival analysis ^27^. The supplementary data tables ( Table S1– Table S7) accompanying this work provides the actual number of flies per experiment and individual results used for analysis.

## Results

**Figure 1.**
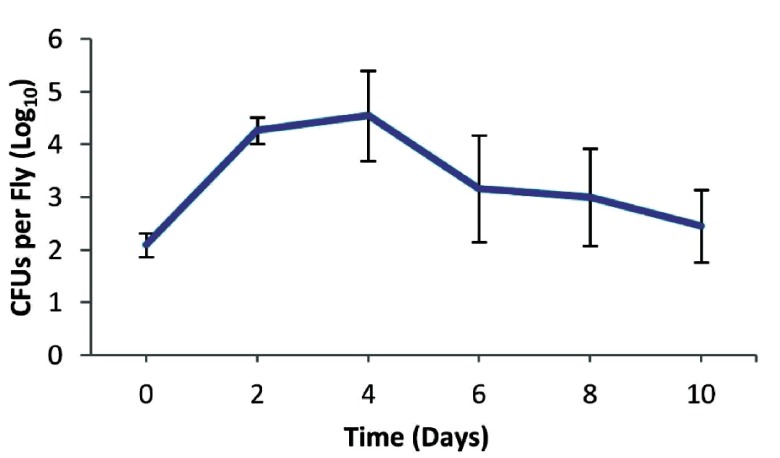
Persistence of *Pseudomonas aeruginosa* CF5 in the fly. Wild type Oregon R male flies were infected with 130 colony forming units (CFUs) of the low virulent *P. aeruginosa* CF5 strain. Infection is persistent for at least 10 days with more than 100 CFUs per fly.

**Figure 2.**
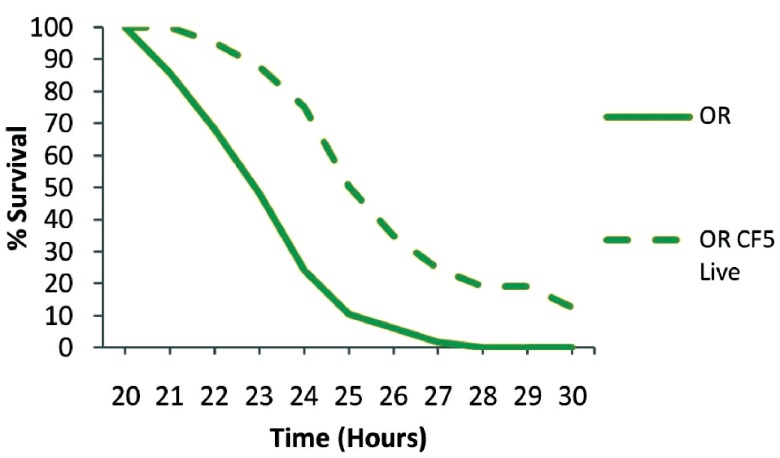
Survival kinetics of wt PA14 infected flies 11 days post priming with live *Pseudomonas aeruginosa* CF5. *P. aeruginosa* PA14 injection in primed (dashed line) and non primed (continuous line) Oregon R flies with live CF5 bacteria showed an extension in the 50% survival time for over of two hours in contrast to controls and more that 10% survived at 30 hours post infection (P<0.0001). n=40–49 flies per condition.

To test if long-term protection could be achieved by immune priming, we initially infected wild type male Oregon R flies with 130 colony forming units (CFUs) of the low-in-virulence *P. aeruginosa* strain CF5. Infection was persistent for at least 10 days when >100 CFUs/fly were still present in the flies ( Figure 1). Injections with the virulent PA14 strain were performed on the 11 ^th^ day of priming with CF5 and in control non-primed flies. In primed flies, the 50% survival time was over two hours longer and over 10% of primed flies had survived at 30 hours post-infection ( Figure 2). It should be noted that *P. aeruginosa* infection with the PA14 strain is reproducibly 100% lethal under these conditions and short time differences between survival curves are biologically and statistically significant using the Kaplan-Meier survival kinetic analysis ^11, 26^. Here we observe a protective role against a virulent bacterium when the host is primed with live bacteria of the same species (P<0.0001) ( Figure 2).

To assess the duration of immune priming when bacteria are not able to replicate and persist in the host, we primed flies with bacteria that were heat-killed for 10 minutes at 60°C. ~3000 heat-killed CF5 cells were injected per fly 2, 5 or 7 days prior to infection with the lethal strain PA14. Under these conditions, no CFUs could be recovered from flies prior to PA14 infection.

**Figure 3.**
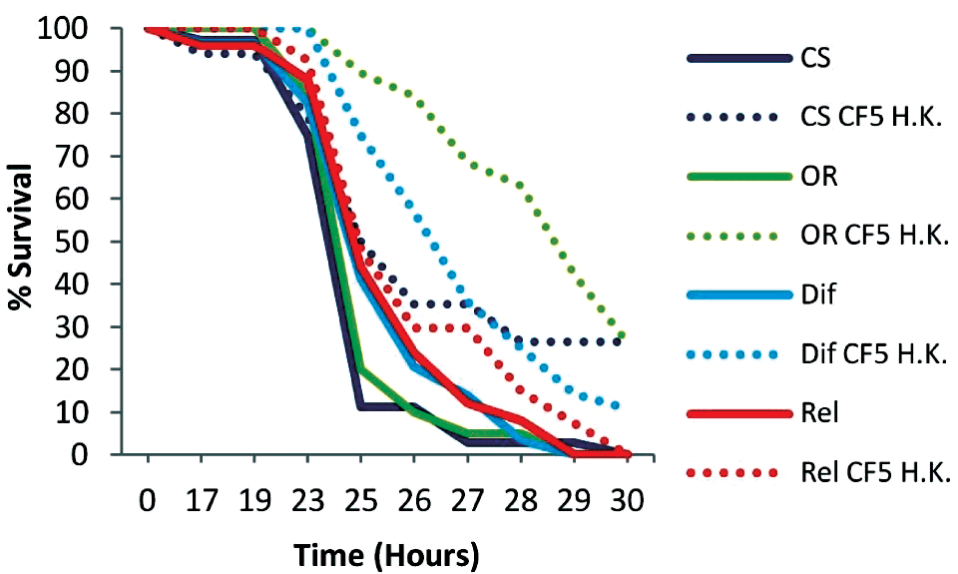
Survival kinetics of wt and humoral response deficient flies 2 days post priming with heat-killed *Pseudomonas aeruginosa* CF5. Wild type Oregon R (OR) and mutant flies of the Imd (Rel) and Toll (Dif) pathways were primed with heat-killed (H.K.) CF5 cells (doted lines) 2 days prior to PA14 challenge. Primed OR flies showed extended survival times and 30% survivors (P<0.0001). Rel mutant control and primed flies died at similar rates indicating a protective role of the *rel* gene (Rel P=0.2199). Dif mutant flies exhibited a low but significant protective effect (P=0.001). n=19–29 flies per condition.

Initially wild type Oregon R (OR), Rel mutant (Imd pathway) and Dif mutant (Toll pathway) mutant flies were primed with heat-killed (H.K.) CF5 cells and infected with the PA14 strain 2 days later. OR flies showed a prominent extension in survival (P<0.0001) with more than 30% of primed flies surviving the infection at 30 hours post-infection ( Figure 3). However the Rel mutant primed and non-primed flies died at similar rates (P=0.2199), suggesting that protective priming responses against *P. aeruginosa* depends on the *rel* gene. Dif mutant flies could elicit a protective response (P=0.0010), which was nevertheless less prominent compared to that of OR flies. Thus *rel* and to a lesser extent *dif*, the 2 main NF-κB immune factors of *Drosophila*, appear to contribute to the protective immune response that lasts for at least 2 days.

**Figure 4.**
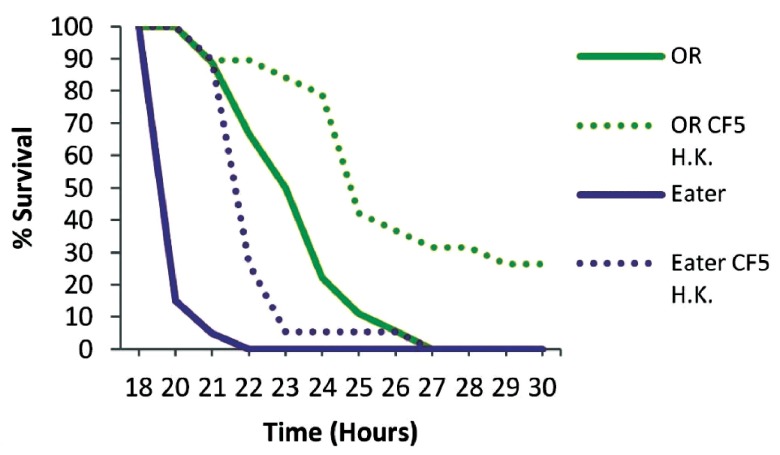
Survival kinetics of wt and cellular response deficient flies 2 days post priming with heat-killed *Pseudomonas aeruginosa* CF5. Eater mutant and wild type Oregon R flies (OR) were primed two days prior to PA14 injection to investigate the role of phagocytosis in immune priming. Eater deficient flies were more susceptible to infection than wild type flies (P<0.0001) in the absence of priming. Nevertheless Eater mutants survived longer when primed indicating additional immune responses of the host at 2 days post priming. n=18–20 flies per condition.

**Figure 5.**
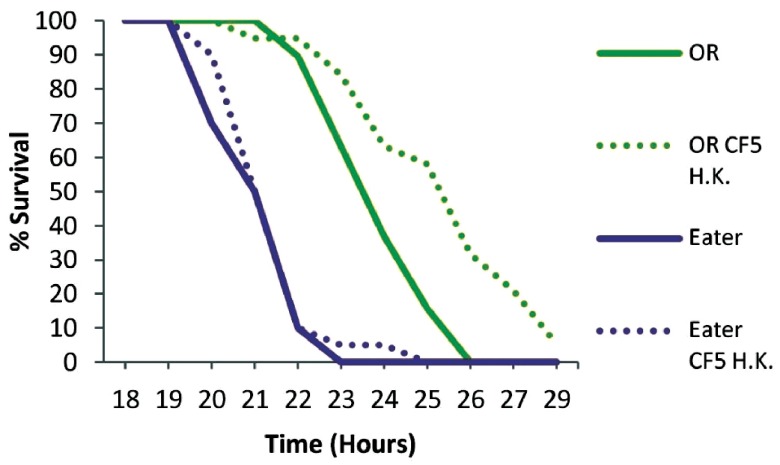
Survival kinetics of wt and cellular response deficient flies 5 days post priming with heat-killed *Pseudomonas aeruginosa* CF5. Wild type Oregon R (OR) and Eater mutant flies where challenged with PA14 5 days post injecting with heat-killed CF5. Eater mutants were equally susceptible to non primed flies (P=0.5616) in contrast to control OR flies (P=0.0037). This apparent cellular response suggests that phagocytosis efficacy depends on the priming period prior to challenge. n=19–20 flies per condition.

To examine if phagocytosis is important for immune priming against *P. aeruginosa* we primed Eater deficient flies for 2 days. Mutant Eater non-primed flies were more susceptible to infection than OR non-primed flies (P<0.0001), and contrary to wild type flies, priming of mutant flies did not lead to any survivors at 30 hours ( Figure 4). Nevertheless, primed Eater deficient flies survived longer when primed (P<0.0001), suggesting that additional immune responses contribute to host protection at 2 days post priming. The role of cellular responses in immune priming was nevertheless clear when the PA14 strain was injected 5 days after priming, when primed Eater mutant flies are equally susceptible to non-primed flies (P=0.5616), while wild type flies were still significantly protected by priming (P=0.0037) ( Figure 5). This suggests that immune priming at 5 days depends heavily on phagocytosis.

**Figure 6.**
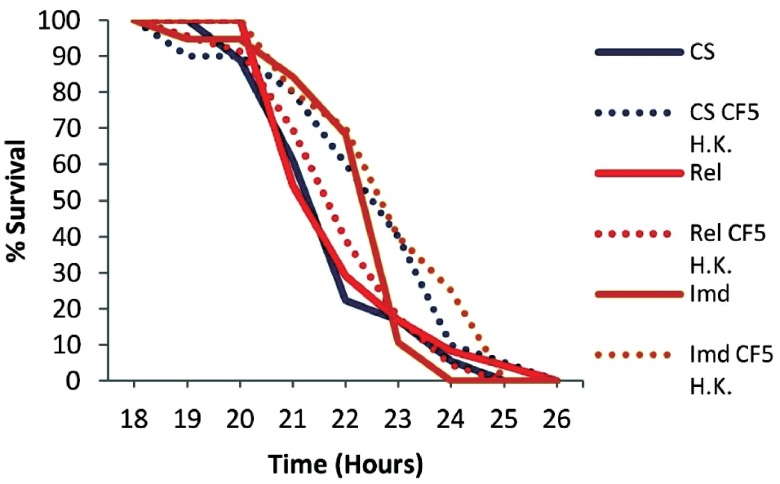
Survival kinetics of wt and humoral response deficient flies 7 days post priming with heat-killed *Pseudomonas aeruginosa* CF5. Wild type Canton S (CS), Imd and Rel mutant primed and non primed flies were tested. All fly genotypes exhibited no significant effect, CS (P=0.0726), Rel (P=0.9163), Imd (P=0.0663), indicating that priming is transient. n=18–24 flies per condition.

**Figure 7.**
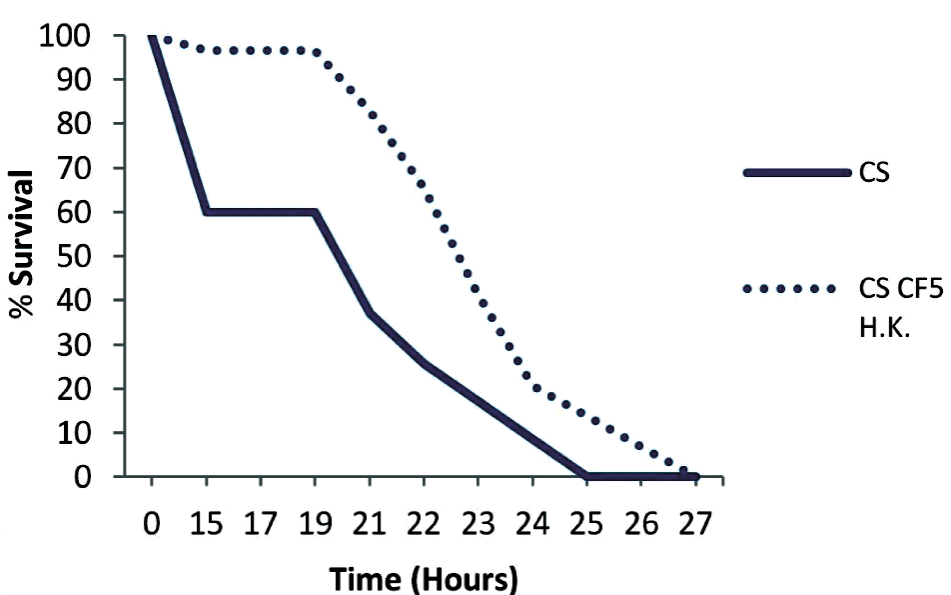
Survival kinetics of wt flies 5 and 7 days post priming with heat-killed *Pseudomonas aeruginosa* CF5. Wild type Canton S flies (CS) were primed with heat-killed CF5 cells 5 and 7 days before *P. aeruginosa* PA14 injection. A protective response was observed (P=0.0007) in the primed flies. n=29–35 flies per condition.

To assess if the priming effect can last for longer, we primed wild type but also Imd and Rel mutant flies 7 days prior to PA14 infection, and we noticed that priming had no significant effect in the survival rates of any of the genotypes tested: Canton S (P=0.0726), Rel (P=0.9163), Imd (P=0.0663) ( Figure 6). To assess if 7 day primed flies are incapable of mounting a protective immune response or if priming diminishes after 7 days, we double primed wild type flies 5 and 7 days prior to PA14 infection. We noticed that double priming extends the survival of flies (P=0.0007) ( Figure 7), thus priming with dead *P. aeruginosa* has a short-lasting protective effect of less than a week.

## Discussion

### Specific responses to different microbes

Collectively our data indicate that low-in-virulence *P. aeruginosa* can prime the *Drosophila* humoral and cellular immune responses against a subsequent lethal infection with a more virulent strain. Nevertheless, unlike priming with *S. pneumoniae* or *B. Bassiana*, this is not a long-lasting effect. It is thus pivotal that future studies assess in detail the differences in the immune responses among many different microbes and in time points that last for many days rather than hours as is customary. Long-term responses to single or repeated challenges of the immune system might pinpoint novel aspects of immunological memory. One aspect of immune responses that might be related to immunologic memory in invertebrates is specificity. A recent breakthrough in the specificity of immune responses in insects came with the discovery of the multi-variable gene *Dscam* ^28^. Dong and his team found that different immune elicitors in the mosquito direct the production of pathogen-specific splice variants of the Down Syndrome Cell Adhesion Molecule receptor necessary for the protection of the host from infection with *Plasmodium*. Though no experiments were done to test the duration of this specific response, this work illustrates the adaptability of the insect immune system. There are additional examples of specific immune responses in invertebrates such as the snail *Biomphalaria glabrata*, in which fibrinogen related proteins (FREPS) exhibit a high rate of diversification at a genomic level, and the expression profiles of the scavenger receptor cysteine-rich proteins in the sea urchin, although how these proteins respond to re-challenge is not known ^29, 30^. Protection against a secondary infection is also seen in the mealworm beetle, *Tenebrio molitor* ^31^. Prolonged protection was observed when initial exposure to lipopolysaccharides (LPS) before infection with spores of the entomopathogenic fungus *Metarhizium anisopliae* occurred. This was attributed to a long-lasting antimicrobial response of the LPS-challenged larva, which provided a survival advantage when it was exposed to fungal infection. Thus invertebrate hosts can be further studied to understand the parameters of long-lasting immune responses and their relation to immune specificity and memory.

In conclusion, the area of immunological memory remains elusive in the invertebrate world and only recently have small steps been made to investigate this aspect of the immune system. Specific responses can occur against particular pathogens. Generalisations on the defence mechanisms do not represent the true complexity of the immune system. Therefore, the immune system of invertebrates is still a field that can advance our understanding of how organisms defend themselves from intruders.

## Figures and Tables

**Table S1.** Persistence of *Pseudomonas aeruginosa* CF5 in the fly. Infected flies were ground and plated, 3 individuals per replicate, at days 0, 2, 4, 6, 8 and 10 post infection where the colony forming units (CFU) were measured and calculated per fly. The experiment was performed in triplicates where the average CFU numbers were plotted with the corresponding standard deviation.

Infection with live CF5											
Time (Days)	0 days	2 days	4 days	6 days	8 days	10 days
CFUs per Fly	220	32000	320000	7000	10000	1330
	100	20000	20000	4500	700	300
	87	10000	7000	100	150	56
Average CFUs per Fly	135,67	20666,67	115666,7	3866,67	3616,67	562
Standard deviation	73,33	11015,14	177077,2	3493,33	5534,97	676,2

**Table S2.** Survival kinetics of wt PA14 infected flies 11 days post priming with live *Pseudomonas aeruginosa* CF5. Oregon R (OR) uninfected flies (n=49) and OR infected with live CF5 bacteria (n=40) for 11 days were injected with the virulent PA14 strain. Top row numbers indicate the time in hours post injection whereas lower rows the corresponding number of surviving flies in naive and primed flies respectively. Sample size is indicated at 20h.

11 Days infected with live CF5											
Time (Hours)	20h	21h	22h	23h	24h	25h	26h	27h	28h	29h	30h
OR	49	42	33	23	11	5	3	1	0	0	0
OR CF5 live	40	40	38	35	30	20	14	10	8	8	5

**Table S3.** Survival kinetics of wt and humoral response deficient flies 2 days post priming with heat-killed *Pseudomonas aeruginosa* CF5. Wild type Oregon R (OR) and mutant Dif and Rel flies were primed with heat-killed (H. K.) CF5 cells. Naive and primed flies were injected with the virulent PA14 strain after 2 days. Top row numbers indicate the time in hours post challenge whereas lower rows indicate the corresponding number of surviving flies in naive and primed flies respectively for each genotype. Sample size is indicated at 0h.

2 Days priming										
Time (Hours)	0h	17h	19h	23h	25h	26h	27h	28h	29h	30h
OR	20	20	20	17	4	2	1	1	0	0
OR CF5 H.K.	19	19	19	19	17	16	13	12	8	5
Dif	29	28	28	24	12	6	4	1	0	0
Dif CF5 H.K.	28	28	28	28	21	16	10	7	4	3
Rel	25	24	24	22	11	6	3	2	0	0
Rel CF5 H.K.	27	27	27	25	13	8	8	4	2	0

**Table S4.** Survival kinetics of wt and cellular response deficient flies 2 days post priming with heat-killed *Pseudomonas aeruginosa* CF5. Oregon R (OR) (n=18) and Eater (n=20) mutant uninfected flies, and primed with heat-killed (H. K.) CF5 bacteria were inoculated at 2 days with the virulent PA14 strain. Top row numbers indicate the time in hours post challenge whereas lower rows indicate the corresponding number of surviving flies in naive and primed flies respectively for each genotype. Sample size is indicated at 18h.

2 Days priming												
Time (Hours)	18h	20h	21h	22h	23h	24h	25h	26h	27h	28h	29h	30h
OR	18	18	16	12	9	4	2	1	0	0	0	0
OR CF5 H.K.	19	19	17	17	16	15	8	7	6	6	5	5
Eater	20	3	1	0	0	0	0	0	0	0	0	0
Eater CF5 H.K.	19	19	17	5	1	1	1	1	0	0	0	0

**Table S5.** Survival kinetics of wt and cellular response deficient flies 5 days post priming with heat-killed *Pseudomonas aeruginosa* CF5. Wild type Oregon R (OR) and Eater mutant uninfected flies (n=19, n=20) and primed with heat-killed (H. K.) CF5 bacteria (n=19, n=20) were inoculated at day 5 with PA14. Top row numbers indicate the time in hours post challenge whereas lower rows indicate the corresponding number of surviving flies in naive and primed flies respectively for each genotype. Sample size is indicated at 18h.

5 Days priming											
Time (Hours)	18h	19h	20h	21h	22h	23h	24h	25h	26h	27h	29h
OR	19	19	19	19	17	12	7	3	0	0	0
OR CF5 H.K.	19	19	19	18	18	16	12	11	6	4	1
Eater	20	20	14	10	2	0	0	0	0	0	0
Eater CF5 H.K.	20	20	18	10	2	1	1	0	0	0	0

**Table S6.** Survival kinetics of wt and humoral response deficient flies 7 days post priming with heat-killed *Pseudomonas aeruginosa* CF5. Wild type Canton S (CS) and mutant Rel and Imd flies were primed with heat-killed (H. K.) CF5 cells. Flies were injected with the virulent PA14 strain 7 days post priming. Top row numbers indicate the time in hours post injection whereas lower rows indicate the corresponding number of surviving flies in naive and primed flies respectively for each genotype. Sample size is indicated at 18h.

7 Days priming									
Time (Hours)	18h	19h	20h	21h	22h	23h	24h	25h	26h
CS	18	18	16	11	4	3	1	0	0
CS CF5 H.K.	20	18	18	16	12	8	2	1	0
Rel	24	24	24	13	7	4	2	1	0
Rel CF5 H.K.	23	22	21	16	9	4	1	0	0
Imd	19	18	18	16	13	2	0	0	0
Imd CF5 H.K.	20	20	20	16	14	8	5	0	0

**Table S7.** Survival kinetics of wt flies 5 and 7 days post priming with heat-killed *Pseudomonas aeruginosa* CF5. Canton S (CS) wild type flies were primed twice with heat-killed (H. K.) CF5 cells at days 5 and 7 prior to injection with PA14. Naive (n=35) and primed (n=29) flies were inoculated with the virulent PA14 strain 7 days post priming. Top row numbers indicate the time in hours post challenge whereas lower rows indicate the corresponding number of surviving flies in naive and primed flies respectively. Sample size is indicated at 0h.

5+7 Days priming													
Time (Hours)	0h	15h	17h	19h	21h	22h	23h	24h	25h	26h	27h	28h	36h
CS	35	21	21	21	13	9	6	3	0	0	0	0	0
CS H.K. CF5	29	28	28	28	24	19	12	6	4	2	0	0	0
