# Supplementary material for: Drosophila immune priming against Pseudomonas aeruginosa is short-lasting and depends on cellular and humoral immunity
Source: F1000Res. 2013 Mar 5;2:76. [Version 1] doi: 10.12688/f1000research.2-76.v1 (PMC3752738; doi:10.12688/f1000research.2-76.v1)
Supplement: Peer review history

# Peer review: Referee response for version 1

**Published:** 2013-03-22

**Authors:** Bruno Lemaitre

**Affiliations:** École Polytechnique Fédérale de Lausanne, Lausanne, Switzerland

This is a short paper on the influence of priming on the *Drosophila* immune response.The results are worthy enough to be published but require major improvements.

1) The paper, especially the introduction, is poorly written. I just mention few problems

*All the parts on the vertebrate adaptive immune should be double checked by an immunologist. Many sentences make no sense.

*Ex*.: 'Cooperation between lymphocytes and antigen presenting cells is the main mechanism of action, according to which naive B lymphocytes expressing a membrane-bound antibody molecule are activated when they bind to their specific antigen and divide quickly into memory B cells and effector B cells that induce humoral immunity'

This is a short cut. B cells are activated upon direct recognition of antigen and a signal coming from T cell that has been previously activated by a DC This is not a mechanism of action... 

• **Intro part on *Drosophila***

- (ref 11) is a paper unrelated to what it is linked ('homeostatic factors').

- Can melanization be considered as a part of the humoral response?

- What does 'captivation' mean?

- The role of dScam in phagocytosis is poorly established compared to NimC1. I am not aware that Eater has been shown to 'locate' bacteria. 

- 'also exhibit local immunity': 'Also' seems weird when the part above discusses systemic immunity.

• **Intro part on specificity**

- Specificity has been shown. Flies activate adapted immune response to aggressors (ex. Toll/Imd, encapsulation only against parasites). This is not new. What is possibly new is the high degree of specificity.

- What is the meaning of 'selected specificity' and 'selective adaptability'?

- 'They also found similar adaptation': the term adaptation is unclear.

• Boman, in his famous 1972 Nature paper, has already shown that priming with *E. clocae* could protect fly from an infection by *P. aeruginosa*. This could be mentioned.Figure 4: Eater is not a cellular deficient mutant but a phagocytic mutant. Idem for 'humoral response mutant' in figure 6. This is not precise enough.

2) All the experiments in figures 3, 4, 5, 6, 7 should be repeated at least a second time in a way to have an independent repeat and a higher number of flies.

3) The observed effect is not striking: improvement of only 2h. From my point of view, this actually suggests that priming is not very efficient and could be simply explained by the long-lasting effect of AMPs or other immune factors. This hypothesis should be discussed first before evoking dScam, other complex mechanisms or the concept of memory. The memory hypothesis of dSCAM is clearly not needed to explain the observed results. 

4) Mutation should be better described and written in italic. Is there an *eater* mutant or are the authors using a set of deficiencies to generate a mutant?

5) Figure 3: this author has already reported that Toll pathway contributes to host defense against *P. aeruginosa*. In this case, can they explain why *dif* mutant survives better than the the wild-type?

I have read this submission. I believe that I have an appropriate level of expertise to confirm that it is of an acceptable scientific standard, however I have significant reservations, as outlined above.

# Peer review: Referee response for version 1

**Published:** 2013-03-20

**Authors:** Dimitrios Kontoyiannis

**Affiliations:** The University of Texas MD Anderson Cancer Center, Houston, TX, USA

This is an interesting report on a complex and somewhat controversial area (insect immune memory responses) by an investigator who has done nice work in the field. The experimental methods are sufficient, although some methodological issues might create some difficulties in data interpretation;

- The results would be more convincing if isogenic strains of *Pseudomonas* and *Drosophila* were used, as differences are rather small (and possibly biologically non-significant), raising the question of genetic-background influences. In addition, backing up the mortality experiments with bacterial burden data (CFU/gr) or even histopathology in selected differences would make the claims much stronger.
- It would be of interest to have, in addition to heat-killed bacteria, septic injury in the same day as the control. Perhaps priming could occur even by septic injury, in a bacteria-independent factor
- Future experimental direction could be to investigate the effect of different inocula and whether there is a critical minimal threshold of exposure that results in priming.
- Another way to investigate the role, if any, of phagocytosis would be to feed wild flies corticosteroids (see Chamilos G *et al*. PNAS) and evaluate if wild type flies have sluggish priming compared to corticosteroid-unexposed flies.
- I would also analyze survival data of figs 3 and 6 by alternative statistical method in view of the close overlap.

I have read this submission. I believe that I have an appropriate level of expertise to confirm that it is of an acceptable scientific standard, however I have significant reservations, as outlined above.

# Peer review: Referee response for version 1

**Published:** 2013-03-18

**Authors:** Bernard Mathey-Prevot

**Affiliations:** Department of Pharmacology & Cancer Biology, Duke University School of Medicine, Durham, NC, USA

In this manuscript, Christofi and Apidianakis present an extension of their previous work on immune priming with *Pseudomonas aeruginosa*. They focus on the duration of the protective response elicited against the virulent PA14 strain by pre-treating adult *Drosophila* flies with live or heat-killed low-in virulence CF5 bacteria. There are few new insights into the mechanisms by which the protective effect is mediated (involvement of the Toll, Rel and Imd pathways as well as phagocytosis), but the report makes the important point that the duration and extent of priming will vary greatly with the bacteria or fungi used in the experiments, and strongly advocates that future studies into the adaptive characteristics of insect immunity should be carried out over more than a day rather than a few hours as it is commonly done. Careful and extended time-courses will uncover important differences in how priming can lead to significantly diverse responses to infection with one strain of bacteria or another.

**General**: I do have some issues and would like the authors to comment on the following:

- The authors present reasonably performed experiments, but I do have some reservation about the conclusions drawn from their experiments. In particular, I was struck that OR and CS strains show some differences in response to priming (Fig. 3). While the end point is similar at 30h for both strains, they show very distinct survival rates between 25 and 29h. This apparent difference might suggest that different genetic background will play an important role in the priming response. In that regard, I wonder why the OR or CS were chosen as controls, rather than strains that are of the same genetic background than the mutants used in this study. What is also surprising is that the authors chose to ignore the apparent discrepancy between the two strains, and furthermore go on to perform additional experiments where they either chose OR (Fig. 4, 5) or CS (Fig. 6, 7) without really justifying or giving a rationale why they included a particular control strain rather than the other. For consistency sake, it would have been better to select one or the other, or include both stains in each experiment.

- I am probably missing something but I don’t get the logic in Fig 7. The double priming at 5 and 7 days prior to infection is said to extend the protective effect over that of 7 days, but in reality they are looking at flies which had been primed last at 5 days prior the challenge. To me, the correct comparison should have been doubly primed CS flies vs. 5 day primed CS flies, rather than untreated CS. Untreated flies should have been included, but only to serve as a reference.
- One potential explanation for the modest involvement of plasmatocytes and phagocytosis in mediating protection against a challenge with PA14 might be that the initial priming leads to a transient increase in plasmatocytes. It would be nice to have a sense of whether the number of circulating plasmatocytes is increased after priming. I realize that the protection observed in priming is supposed to be strain specific (no cross protection against other bacteria); however, the protective effect related in this report is rather modest. As such, one wonders whether there are two types of responses: 1) The increase in plasmatocytes, which alone would confer a broad protection but be too weak to have a significant effect on other bacteria, and 2) The humoral and recognition pathways that confer specificity against a particular strain.

**Minor:  **The sentence on page 2, starting with “In addition to the plasmatocyte-expressed…” to the end of the paragraph is awkward and needs to be edited.

I have read this submission. I believe that I have an appropriate level of expertise to confirm that it is of an acceptable scientific standard.

# Peer review: Referee response for version 1

**Published:** 2013-03-12

**Authors:** Petros Ligoxygakis, Marcus Glittenberg

**Affiliations:** Department of Biochemistry, University of Oxford, Oxford, UK; Department of Biochemistry, University of Oxford, Oxford, UK

In this manuscript, the authors study a controversial issue in insect immunity: the existence of memory or in other words whether sub-lethal doses of a pathogen may “prime” the insect to respond more efficiently (and survive) a subsequent infection with an otherwise lethal dose of the same pathogen.

Memory in insect immunity has been reported since the field began in the classic work of Metalnikow [Metalnikow S (1929) Immunité d’adaptation et immunité de defense SR. *Soc. Biol.* **101**, 34–67] and more recently in a paper by David Schneider’s lab [Pham LN, Dionne MS, Shirasu-Hiza M, Schneider D (2007) A specific primed response in Drosophila is dependent on hemocytes. *PLoS Pathog*. **3**, e26] as well as in a paper published by Siva-Jothy’s lab in Sheffield [Moret Y & Siva-Jothy MT (2003). Adaptive innate immunity? Responsive-mode prophylaxis in the mealworm beetle, *Tenebrio molitor*. *Proceedings of the Royal Society of London B*, **270**: 2475-2480].

Therefore, the possible existence of memory (or “priming”) is an interesting subject and warrants further investigation to explore the limitations of such a response and its characteristics. The types of experiments that one would use to do this (and are indeed used in this work) are mainly survival experiments following infection. Any differences must be very well documented with appropriate statistical tests.  

Regarding statistical analysis one comment that I would like to make (prompted by Figure 6; curves for other figures seem OK) is that crossing survival curves between different treatments/genotypes indicate non-proportional hazards (so one needs to check for crossing hazards to be sure). Such a scenario increases the probability of a Type II error when using the log-rank test (and weighted log-rank tests) i.e. concluding there is no statistical difference when there actually is one. **There are alternative tests to analyse data with crossing hazards (e.g. Renyi-type), and applying one of these to the data set of Figure 6 in addition to their current analysis is a must.**

Regardless of statistics, their wild-type-background result (and the basis of the research) is a shift in survival of 2 – 4 hours if flies are pre-primed: what is the significance of that? For Figures 3 and 4 the OR flies survive at approx. 30% by 30 hours (although I am not clear of how many times these experiments were repeated? See below comment) – **what would be of more interest is whether these flies have recovered from the PA14 infection** i.e. do they continue to survive after 30 hours (and for how long)? Have the flies cleared / controlled the PA14 bacteria. **The authors therefore need to look at survival and CFUs beyond 30 hours.**

Further, the biggest shift at 50%_survival - from all the experiments - between primed and non-primed is 4 hours (Fig. 3, OR): **is this a real difference or is it within the boundaries of variation?** Generally, from what I can tell, experiments have only been performed once, and mostly this is with 19 – 35 flies: **if more flies were used, along with biological repeats, would the same trends consistently be observed?**

Finally, I think the authors really need to show the fly survival with CF5 and heat-killed CF5; but more importantly, inject live CF5 into the pre-seeded flies as they did with PA14 i.e. if then, the pre-seeded CF5-live flies survive slightly better as they did with PA14, it points to a general mechanism rather than one relating to the virulence of the bacteria. And vice versa, where if a strain is more virulent, then “priming” may be advantageous. 

Minor: Generally, data is better presented as Kaplan-Meier curves; since you are dead or not dead, having slopes between time-of-death is misleading (unless the data set is modelled).In figure legends: if error bars are shown, it should be indicated what they are describing. And the test used to derive the P-value should be given with this value.

We have read this submission. We believe that we have an appropriate level of expertise to confirm that it is of an acceptable scientific standard, however we have significant reservations, as outlined above.
